# Artisanal Goat *Coalho* Cheese with Cachaça as Flavoring Agent

**DOI:** 10.3390/foods12101945

**Published:** 2023-05-10

**Authors:** Iuri Lima dos Santos Rosario, Carla Paulo Vieira, Luana Sipaúba Moreno Barreto, Nathália Brizack Monteiro, Rodrigo Vilela de Barros Pinto Moreira, Ana Paula Salim, Carini Aparecida Lelis, Manuela da Silva Solca, Sergio Borges Mano, Carlos Adam Conte-Junior, Marion Pereira da Costa

**Affiliations:** 1 Graduate Program in Veterinary Hygiene (PPGHIGVET), Faculty of Veterinary Medicine, Fluminense Federal University (UFF), Vital Brazil Filho, Niterói 24220-000, RJ, Brazil; rodrigovilela@id.uff.b (R.V.d.B.P.M.); sergiomano@id.uff.br (S.B.M.); conte@iq.ufrj.br (C.A.C.-J.); marioncosta@ufba.br (M.P.d.C.); 2 Laboratory of Inspection and Technology of Milk and Derivatives (LaITLacteos), School of Veterinary Medicine and Zootechnics, Federal University of Bahia (UFBA), Ondina, Salvador 40170-110, BA, Brazil; luanavet07@gmail.com (L.S.M.B.); nathaliabrizack@hotmail.com (N.B.M.); 3 Center for Food Analysis (NAL), Technological Development Support Laboratory (LADETEC), Federal University of Rio de Janeiro (UFRJ), Cidade Universitária, Rio de Janeiro 21941-598, RJ, Brazil; carlavieira@edu.unirio.br (C.P.V.); apaula15br@yahoo.com.br (A.P.S.); carinilelis@yahoo.com.br (C.A.L.); 4 Analytical and Molecular Laboratorial Center (CLAn), Institute of Chemistry (IQ), Federal University of Rio de Janeiro (UFRJ), Cidade Universitária, Rio de Janeiro 21941-909, RJ, Brazil; 5 Laboratory of Advanced Analysis in Biochemistry and Molecular Biology (LAABBM), Department of Biochemistry, Federal University of Rio de Janeiro (UFRJ), Cidade Universitária, Rio de Janeiro 21941-909, RJ, Brazil; 6 Graduate Program in Animal Science in the Tropics (PPGCAT), School of Veterinary Medicine, Federal University of Bahia (UFBA), Ondina, Salvador 40170-110, BA, Brazil; manuela.solca@ufba.br; 7 Graduate Program in Food Science (PPGCAL), Institute of Chemistry (IQ), Federal University of Rio de Janeiro (UFRJ), Cidade Universitária, Rio de Janeiro 21941-909, RJ, Brazil; 8 Graduate Program in Sanitary Surveillance (PPGVS), National Institute of Health Quality Control (INCQS), Oswaldo Cruz Foundation (FIOCRUZ), Rio de Janeiro 21040-900, RJ, Brazil; 9 Graduate Program in Chemistry (PGQu), Institute of Chemistry (IQ), Federal University of Rio de Janeiro (UFRJ), Cidade Universitária, Rio de Janeiro 21941-909, RJ, Brazil

**Keywords:** caprine cheese, growth modeling, small-scale producers, family farming, sensory acceptability, food preference

## Abstract

Cachaça is a traditional Brazilian drink that has the potential to offer a new sensory and technological strategy for artisanal cheesemaking, particularly for small-scale producers and family farming. This study aimed to investigate the effects of cachaça immersion on the physicochemical, microbiological, color, texture, and sensory parameters of artisanal goat *coalho* cheeses using three different varieties of cachaça immersion. The results showed that cachaça immersion did not affect the cheese’s proximate composition or starter culture viability, indicating its suitability as a new method for artisanal cheese production. Additionally, gold cachaça aged in oak casks was the most effective variety for sensory acceptance and purchase intention, suggesting that it may be a valuable strategy for small-scale producers to add value and encourage the consumption of artisanal goat *coalho* cheeses without compromising their quality. Thus, this study provides important insights for small-scale producers and family farming to enhance their product offerings and increase their competitiveness in the market.

## 1. Introduction

Unlike mass-produced cheeses, artisanal cheeses refer to products that are handcrafted and made in small batches, allowing for greater control over the flavor and texture of a final product that is unique and distinctive [1]. As the popularity of artisanal cheeses continues to grow, there has been a growing interest in locally sourced products, which has increased the number of small-scale cheesemakers who are enthusiastic about producing cheeses using traditional techniques and high-quality ingredients [2]. In this context, with the significant growth in Brazilian dairy goat farming in recent years, particularly in the Northeast region which holds the largest herd of goats (95%) [3], there has been an increase in the availability and diversity of goat milk products in the market. Thus, the growth in goat farming has not only improved small producers’ earnings but has also allowed for the development of new and innovative goat milk products to cater to the expanding market demand [4]. This can be attributed to several factors, including the growing interest in alternative and healthier food options and the increasing awareness of the nutritional benefits of goat milk products [5,6]. As a result, consumers can now enjoy a broader range of goat milk products, such as cheeses, yogurts, and ice creams, that were previously not as readily available [7].

Given the current market demand for a wider variety of goat milk products, including artisanal and small-scale production, there is a need to increase the diversity of these products to attract non-habitual consumers and expand the demand for goat dairy products [7,8]. By offering a greater range of unique and high-quality goat milk products, artisanal cheesemakers can increase the appeal of these products to a broader audience, thereby helping to support the growth of family farming and creating new market opportunities. Among the various popular kinds of Brazilian cheese, goat *coalho* cheese is a typical semi-hard product widespread in the country, mainly in the Northeast region, presenting high social, economic, and cultural value [9]. The cheese is produced from the enzymatic coagulation of milk, consisting of semi-hard, white-colored cheese with a typically opened texture, with mechanical eyes, and a salty, slightly acidic flavor, addressed to the direct consumption market [10]. Improvements to promote greater diversity of goat *coalho* cheeses in the market have been attempted, such as with the addition of probiotics [11,12,13] or aromatic herbs extract [14], and with immersion in alcoholic beverages [15]. In this context, a potential sensory strategy could be immersing cheese in alcoholic beverages, particularly those with widespread acceptance, such as cachaça.

Cachaça is a genuine, highly consumed Brazilian spirit, representing 87% of the national market and one of the four most consumed beverages worldwide [16]. According to Brazilian legislation [17], there are different varieties of cachaça: (1) aged, when 50% of its content is stored in a wooden cask with a maximum capacity of 700 L for at least 1 year, or (2) unaged, when bottled after distillation and stored in wood/steel containers for a period shorter than 1 year, in casks larger than 700 L. Both cachaças can be classified as gold (yellow) or silver (white) cachaça, depending on the final color [18]. Additionally, as cachaça has an alcohol percentage content of between 38 and 48%, its strong taste might soften the characteristic goaty flavor and aroma of the cheese, thus making goat products more pleasant for those consumers unfamiliar with them. Therefore, artisanal goat *coalho* cheeses immersed in cachaça can be an innovative strategy for small-scale cheesemakers and family farming.

Family farming plays a crucial role in food production, particularly in many rural areas. However, small-scale farmers often face challenges in finding a product market and may struggle to compete with larger, industrialized producers [5]. Small-scale cheesemakers can differentiate themselves and potentially reach a new market by creating unique products, such as artisanal cheeses immersed in cachaça. Moreover, adding value to a product can increase profitability for small-scale farmers [19]. By using traditional methods and adding cachaça, these artisanal goat cheeses can command higher prices than traditional goat *coalho* cheeses. This can provide small-scale farmers with additional income and potentially enable them to expand their operations. Finally, producing artisanal goat cheeses immersed in cachaça can help preserve traditional cheesemaking methods and promote a region’s cultural heritage, a trend already happening in different parts of the world [20]. This can create a sense of identity and pride for small-scale cheesemakers, which can be important for their sense of community and well-being. In summary, artisanal goat *coalho* cheeses immersed in cachaça can provide small-scale cheesemakers with a unique and profitable product to help them compete in a crowded market, preserve traditional methods, and promote cultural heritage. However, while numerous studies have examined various methods of cheese production, the effects of the immersion of cheeses in cachaça have been understudied, with only a select few experiments having explored its potential impact [15,21]. Therefore, this study aimed to evaluate the effect of cachaça immersion (gold cachaça aged in oak casks, gold cachaça stored in balsam casks, and silver cachaça) on the microbiological, color, textural, and sensory parameters of artisanal goat *coalho* cheeses during 60 days of storage at 4 °C.

## 2. Materials and Methods

100 L of raw whole goat milk (Saanen and Toggenburg breeds) was acquired from Kadosh Ranch (Camaçari, Bahia, Brazil; −12.811542243799714, −38.28553979174119). Additionally, 0.01% (*w*/*v*) mesophilic culture (*Lactococcus lactis* subsp. *lactis* and *Lactococcus lactis* subsp. *cremoris*, R-704^®^, Chr. Hansen, Valinhos, Brazil), 0.05% (*v*/*v*) calcium chloride, 0.005% (*v*/*v*) rennet (Chy-max EXTRA^®^, Chr. Hansen, Valinhos, Brazil), and 1.5% (*w*/*v*) NaCl were used, respectively, for acidification, coagulation, and salting during the process of cheese manufacturing. The ingredients were obtained from local supermarkets.

### 2.1. Goat Coalho Cheese Production

The traditional artisanal goat *coalho* cheese production procedure was modified from BR Patent No. 1020200168860 [22]. Briefly, the milk was pasteurized (65 °C for 30 min), cooled to 35 °C, and the starter culture, calcium chloride, and coagulant was added. Then the cheese manufacture took place normally until the molding step, when the cheeses were pressed for 4 h. Overall, the cheeses were ripened at 11 °C for 8 days. Nevertheless, on the fourth day of ripening, the cheeses were weighed and subdivided into four groups, totalizing approximately 2.5 kg of cheese for each group. One group returned to the maturation chamber without immersion (plain cheese). At the same time, the other three cheese groups were immersed in containers with different types of cachaças (Ypioca^®^, Fortaleza, Brazil; purchased at a local supermarket in Salvador, BA, Brazil) and immediately returned to the maturation chamber, where the immerged cheeses were kept for a total of 8 h. After this time, the cheeses were removed from the cachaça immersion and replaced in the maturation chamber until the end of the ripening process. The immersion technique allowed cheeses to absorb the flavors and aromas of cachaça without any liquid loss caused during milk coagulation. Finally, this approach allowed greater control over the infusion process, enabling us to precisely regulate the amount of alcoholic beverage used and the duration of the immersion. Therefore, a total of four treatments of artisanal goat *coalho* cheese were performed: (1) unflavored, to serve as the control (GC); (2) immersed in gold cachaça aged in oak casks (GCGO); (3) immersed in gold cachaça stored in balsam casks (GCGB); and (4) immersed in silver cachaça (GCS). After ripening, the cheeses were packed, sealed in polyethylene plastic bags, and stored at 4 °C for 60 days, which ensured a margin of reliability for evaluating the physicochemical, microbiological, and instrumental aspects of the cheese. All analyses were conducted in triplicate (*n* = 3). The pH, microbiological, color, and textural determinations occurred on days 0, 10, 20, 30, 40, 50, and 60 of storage.

### 2.2. Proximate Composition, Water Activity, and pH

Moisture and dry matter content were analyzed by infrared radiation drying (model i-Thermo G 163L, BEL Engineering^®^, Monza, Italy) according to the official method (AOAC, 2016) and expressed in g/kg. Fat content (g/kg) was determined using the Gerber method described in the official method (AOAC, 2016), where samples were combined with sulfuric acid and amyl alcohol, followed by centrifugation, and measured by a cheese-calibrated butyrometer. Afterwards, fat in dry matter (FDM) was obtained by dividing fat by dry matter multiplied by 100, all in agreement with the official AOAC methods [23]. The water activity (a_w_) was measured using an AQUALAB Pawkit METER (Decagon Devices^®^, Washington, DC, USA). Proximate composition was determined on day 0 of storage, while a_w_ was determined on days 0 and 60.

The pH of cheeses was measured with a digital potentiometer (model 720 P, ISTEK, Seoul, Republic of Korea) equipped with a DME-R12 electrode (Digimed^®^, São Paulo, Brazil) by direct insertion into a solution consisting of 10 g of each cheese and 90 mL of distilled water, according to the AOAC method [23]. Before use, the electrode was calibrated with standard buffer solutions of pH 4.00 and 7.00.

### 2.3. Bacterial Enumeration and Growth Modelling

At selected time intervals during the 60-day storage period, three cheese samples (10 g) were individually homogenized and serially diluted in 90 mL of 0.1% peptone water in a stomacher (Stomacher 80, Seward, London, UK), followed by plating in two different media. For *Lactococcus* spp. counts, different aliquots were plated in M17 agar, supplemented with lactose, and aerobically incubated at 35 °C for 18–24 h. For LAB counts, aliquots were plated in MRS agar and aerobically incubated at 37 °C for 48–72 h, according to APHA (2015). The enumeration of colonies was performed using an electronic counter (Flash & Go, IUL instruments, Barcelona, Spain) after incubation of each bacterium, and results were expressed as log colony forming units (CFU) per gram.

Each growth curve was constructed separately from a single replicate by fitting the experimental data to the primary model developed by Baranyi and Roberts [24] (Equations (1)–(3)) using the DMFit Excel add-in package version 3.5 (ComBase, United States Department of Agriculture-Agricultural Research Service, Washington, DC, USA). As a result, the estimated kinetic parameters were reported as the maximum growth rate (*μ_max_*), initial cell concentration (*y*_0_), lag phase (λ), and maximum cell concentration (*y_max_*):

(1) $$y\left(t\right)=y_{0}+\mu_{max}A\left(t\right)-\frac{1}{m}\ln\left(1+\frac{e^{m\mu_{{max}^{A\left(t\right)}}}-1}{e^{m(y_{max-y_{0}})}}\right)$$

(2) $$A\left(t\right)=t+\frac{1}{\mu_{max}}\ln\left(\frac{e^{{-\mu }_{{max}^{t}+q_{0}}}}{1+q_{0}}\right)$$

(3) $$\lambda =\frac{\ln\left(1+\frac{1}{q_{0}}\right)}{\mu_{max}}$$

where: *y*(*t*) = population of cells at time t (log CFU/g); *y*_0_ = initial cell concentration (log CFU/g); *μ_max_* = maximum specific growth rate (log CFU/g/h); *y_max_* = maximum cell concentration (log CFU/g); *q*_0_ [-] = parameter that expresses the initial physiological state of the cells; λ = lag phase time (h).

### 2.4. Color Determinations and Texture Analysis

Color determinations such as lightness (L*, 100 = white, 0 = black), redness (a*, +red, -green), and yellowness (b*, +yellow, -blue) were carried out at 10 °C using a Minolta CM-600D spectrophotometer (Minolta Camera Co., Osaka, Japan) according to İncedayi [25]. The color parameters were determined at two random locations on each cheese’s surface immediately after removing the packaging. Additionally, chroma (C*), hue angle (*h°*), and total color difference (ΔE*) were calculated based on the analyzed color coordinates, where the ΔE* was calculated matching the spectrum of the freshly prepared cheeses (day 0) and their relative spectrum at the subsequent storage days (days *n* = 10 to 60), as follows (Equations (4)–(6)):

(4) $$C^{*}={\left({a^{*}}^{2}+{b^{*}}^{2}\right)}^{\frac{1}{2}}$$

(5) $$h{}^{\circ}=\arctan\left(\frac{b^{*}}{a^{*}}\right)$$

(6) $$\Delta E=\sqrt{{\left(L_{n}^{*}-L_{0}^{*}\right)}^{2}+({a_{n}^{*}-a_{0}^{*})}^{2}+({b_{n}^{*}-b_{0}^{*})}^{2}}$$

Hardness (*g*), cohesiveness (*g*), gumminess, and chewiness were determined by texture profile analysis (TPA) using a texture analyzer (TA-XT.Plus, Stable Micro Systems Ltd., Godalming, UK). Samples were cut into cubes (1 cm × 1 cm × 1 cm) and two measurements were made on three different cheese samples at room temperature using a cylindrical aluminum probe (P/36 R, 3.6 cm diameter). The results were analyzed using Texture Expert software version 2.64 (Stable Micro Systems Ltd., Godalming, UK).

### 2.5. Acceptance and Purchase Intention

Sensory evaluation was conducted using 118 untrained panelists (83 women, 35 men), aged 18 to 56 years. Consumers were randomly selected at the Federal University of Bahia, Brazil. The inclusion criterion was the regular consumption of dairy products, and individuals reporting milk allergy and lactose intolerance were not included in the panel. Before the evaluation session, the participants were briefed on how to evaluate the cheese samples to better understand the parameters to be evaluated. In addition, the panelists received filtered water and unsalted crackers to cleanse their palate between cheese samples. Along with each sample, the panelists received a form containing an acceptance test where the participants evaluated appearance, color, aroma, flavor, texture, and overall impression on a 9-point hedonic scale (1 = extremely dislike to 9 = extremely like). Further, to evaluate purchase intention, panelists responded to a 5-point scale (1 = definitely would not buy, 3 = might buy/might not buy, and 5 = definitely would buy).

### 2.6. Statistical Analysis

The results were reported as the mean ± standard deviation (SD). The assumption of normality of the quantitative variables was assessed by the Shapiro-Wilk test (*p* < 0.05). The proximate composition, microbiological, color, texture, and sensory results were assessed by analysis of variance (one or two-way ANOVA) at 0.05 significance level using the software XLSTAT version 2022.1 (Addinsoft, Paris, France). Measures of the coefficient of determination (R^2^) were used to evaluate the performance of the primary growth models built in this study using the Microsoft Office Excel software version 2016 (Microsoft Corporation, Washington, US). Finally, GraphPad Prism version 8.0.0 (GraphPad Software, San Diego, CA, USA) was used to plot all figures and evaluate the linear correlation between the variables using Pearson’s correlation test with a significance level of 0.05.

## 3. Results and Discussion

### 3.1. Moisture, Fat in Dry Matter, Water Activity, and pH of Cheeses

**Figure 1:**
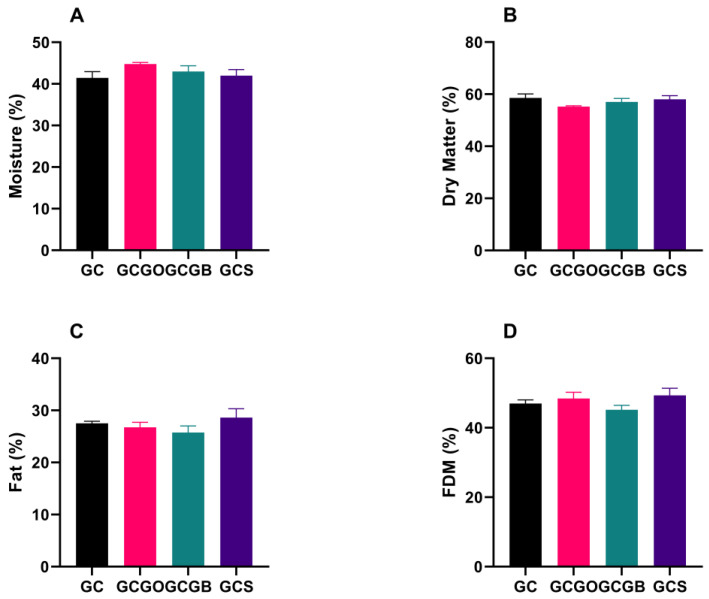
Compositional parameters (means ± standard deviation) of artisanal goat *coalho* cheeses immersed in cachaça on day 0 of storage. (**A**) Moisture; (**B**) Dry matter; (**C**) Fat; (**D**) FDM = Fat in dry matter. GC = artisanal unflavored goat *coalho* cheese; GCGO = artisanal goat *coalho* cheese immersed in gold cachaça aged in oak barrels; GCGB = artisanal goat *coalho* cheese immersed in gold cachaça aged in balm barrels; GCS = artisanal goat *coalho* cheese immersed in silver cachaça.

The moisture and fat in dry matter (FDM) of the artisanal goat *coalho* cheeses (day 0) are summarized in Figure 1. All cheeses met the parameters prescribed by the Brazilian legislation [26] for moisture content (>36% and <54.9%) and fat content in dry matter (>35% and <60%), confirming that the cachaça immersion is suitable for the production of *coalho* cheese. In addition, no difference (*p* > 0.05) was found in any of the chemical parameters studied (*p* > 0.05), indicating that the cachaça immersion does not affect the moisture and FDM of artisanal goat *coalho* cheeses.

**Figure 2:**
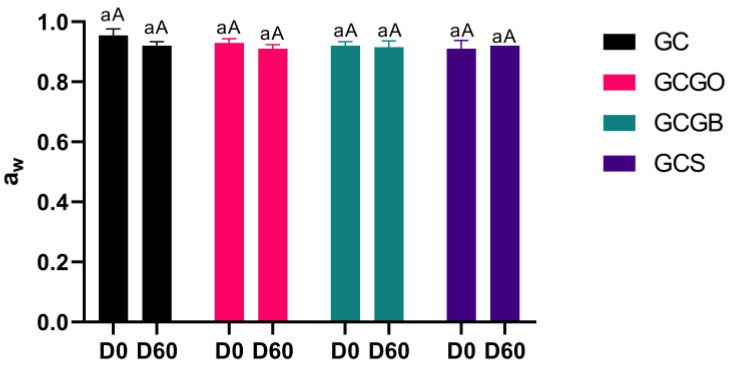
Water activity (means ± standard deviation) of artisanal goat *coalho* cheeses immersed in cachaça on days 0 and 60 of storage. Different lowercase superscripts indicate significant differences regarding storage times (*p* < 0.05), and different uppercase superscripts indicate significant differences regarding treatments (*p* < 0.05); D0 and D60 = days 0 and 60 of storage, respectively.

Similarly, the a_w_ of all cheese samples was in the range of 0.91–0.96, showing no significant differences (*p* > 0.05) between treatments or sampling times (Figure 2). This pattern reinforces that cachaça addition did not affect the samples’ free water loss/gain. Artisanal *coalho* cheese contains an average of 570–1903 (mg/100 g) NaCl included in the salting step. NaCl addition may have increased the stability of the casein network, avoiding the additional whey loss in the immersed treatments during ripening. This fact can be explained because NaCl has been reported to increase the ethanol stability of milk due to an increase in Na/K ratio after Na addition [27].

**Figure 3:**
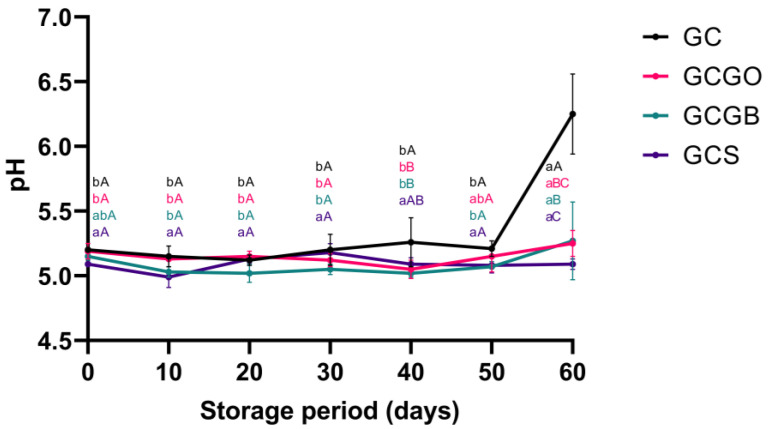
pH (means ± standard deviation) of artisanal goat *coalho* cheeses immersed in cachaça during 60 days of storage. Different lowercase superscripts indicate significant differences regarding storage times (*p* < 0.05), and different uppercase superscripts indicate significant differences regarding treatments (*p* < 0.05).

Further, the changes in pH of the artisanal cheeses during the 60-day storage period are shown in Figure 3. In general, cheese pH remained constant, with similar (*p* > 0.05) values for all treatments until the 30th day of storage. However, GC tended to increase, reaching statistical significance on days 40 and 60 (*p* < 0.05). Indeed, GC tended to present a higher pH than the cachaça-immersed treatments. In fact, the increase in pH after 40 days of storage may have been caused by the breakdown of protein and peptides leading to the accumulation of ammonia and amines, which are alkaline compounds produced by the starter culture during proteolysis [28]. Concerning the cachaça-immersed cheeses, GCGB was the most acidic cheese throughout storage, while GCGO and GCS had intermediate values. Although the current regulation does not set standards for the pH of cachaça [17], this product is considered acidic, with pH values between 3.9 and 5.3, due to the accumulation of organic acids, extraction of acids from wood casks, or oxidation reactions of alcohol and aldehydes [29].

### 3.2. The Effect of Cachaça-Immersion on the Growth Parameters of the Starter Culture

The growth curves of the starter cultures were studied for 60 days-storage at 4 °C, and the primary growth parameters are shown in Table 1. The growth curves of BAL and *Lactococcus* spp. determined in DMFit during artisanal goat *coalho* cheese storage showed a high correlation coefficient (R^2^ ≥ 0.97), indicating a good fit between the experimental data and the model.

All LAB growth curves on artisanal goat *coalho* cheeses started with a similar (*p* > 0.05) initial concentration, presenting an increase of up to 2.01-fold when reaching the final concentration (*p* > 0.05) of about 11 log CFU/g. On the other hand, it was observed that the type of cachaça variety reduced the initial lactococcal concentrations by up to 1.04-fold in GCGB compared to the control, with a significant decrease (*p* < 0.05) by 1.27-fold in GCGO and 1.15-fold in GCS, suggesting a cachaça inhibitory/bacteriostatic effect upon initial contact with *Lactococcus* spp. cells. According to Spano and Massa [30], LAB such as *Lactococcus* spp. possesses stress-response mechanisms, some of which can be associated with acidic pH and low ethanol concentrations, that allow bacteria to survive intense physical and chemical injuries in the cell membrane by preventing disruption and leakage of intracellular compounds. Still, the antimicrobial constituents of cachaça and their antimicrobial interactions require further research in the cheese matrix during maturation/storage. Finally, there were no significant differences (*p* > 0.05), regardless of the treatments, at the end of the storage. Indeed, counts of LAB and *Lactococcus* spp. showed a strong positive correlation with storage time, (r = +0.958, *p* < 0.0001) and (r = +0.932, *p* < 0.0001) respectively (Table S1), confirming the increase in cell numbers during storage and validating the efficiency of incorporating the starter culture in cheese production immersed in cachaça.

The maximum growth rate (concentration of cells in log/gram/hour) and lag phase time (hour) of the developed models were also estimated (Table 1). Both LAB and *Lactococcus* spp. exhibited similar (*p* > 0.05) growth rates, indicating that cachaça did not affect this parameter. On the other hand, LAB exhibited a much shorter lag phase in GC than predicted for GCGB (1.62-fold longer than GC), reaching statistical significance (*p* < 0.05) when compared to GCGO and GCS (4.79 and 4.94-fold longer than GC, respectively), indicating that cachaça had an impact on LAB adaptation in these treatments, especially gold cachaça aged in oak cask and white cachaça. Apart from the aforementioned possible antimicrobial effect, another explanation for this pattern is the lower (*p* < 0.05) initial counts of *Lactococcus* spp. in GCGO and GCS, since *Lactococcus* spp. was the predominant bacteria among LAB during cheesemaking, and LAB showed a strong positive correlation with *Lactococcus* spp. counts (r = +0.937, *p* < 0.0001) (Table S1). Nevertheless, the cachaça immersion can be considered microbiologically suitable for goat *coalho* cheese production since all treatments showed high viability of starter cultures and higher cell counts throughout the 60-day storage period.

### 3.3. Instrumental Color

Color properties of artisanal goat *coalho* cheeses were evaluated through L* (lightness/darkness), a* (redness/greenness), and b* (yellowness/blueness) during 60 days of storage at 4 °C, and the results are exhibited in Table 2. Color is an essential feature in the appearance of cheese as it is perceived during the consumer’s first contact with the product. This parameter contributes to better characterization and standardization of the product manufacturing process, increasing purchase intention [31]. For example, essential attributes for consumers of *coalho* cheese from different regions of Brazil are brightness, and yellow and white color [32].

The parameter L* ranges from black (0) to white (100), where values closer to 100 denote brighter samples. At the beginning of storage (day 0), cachaça-immersion did not affect L* values as no difference (*p* > 0.05) was observed among treatments. Additionally, L* showed a tendency of continuous decrease (*p* < 0.05) in all cheeses during storage, which can be confirmed by the inverse correlation between L* and storage time (r = −0.747, *p* < 0.0001) (Table S1). This decrease can be attributed to proteolysis and casein solubilization, resulting in dark coloration and the concentration of cheese components caused by moisture loss during storage [31]. To corroborate, L* was inversely correlated with LAB (r = −0.733, *p* < 0.0001) and *Lactococcus* spp. (r = −0.723, *p* < 0.0001), which may be attributed to the higher proteolytic activity of these bacteria, resulting in increased pH (Table S1).

After day 30, GC was significantly darker (*p* < 0.05) than the other treatments, up to 1.05-fold, indicating higher proteolysis/dehydration or a possible brightening effect of cachaça during storage. Interestingly, GCGB exhibited higher (*p* < 0.05) L* values than the other treatments on days 10, 20, and 30. The increase in brightness in this treatment may be related to the much higher color intensity of the cachaça stored in balsam cask compared to other variants [33]. Finally, at the end of storage (day 60), all treatments had similar (*p* > 0.05) L*, values that are consistent with other reported studies [34].

The parameter a* ranges from a degree of greenness (from −80 to 0) to redness (from 0 to 100), with values near zero indicating that perceived greenness/redness is low. On day 0, cachaça-immersion increased a* values up to 3.16-fold, while GC had the lowest value (*p* < 0.05). Indeed, cachaça stored in balsam casks is known to have a more robust, reddish color tone, which explains the highest (*p* < 0.05) a* value of this treatment [33]. However, a* progressively decreased during storage in all treatments, especially in the immersed cheeses. The cachaça color is mainly attributed to the tannins released from the wooden casks and their oxidation products. Gallic acid, a product of tannin breakdown, and syringaldehyde are two predominant phenolic compounds in cachaça stored in balsam casks and aged in oak casks [33]. In this context, gallic acid has been reported to act as a copigmentation factor by intensifying the red color perception of red wine [35], while syringaldehyde has been associated with higher red hue perception [36], possibly explaining the elevated a* values in GCGB and GCGO.

On the other hand, the silver cachaça, produced in inert containers, presents slower chemical reactions with minor production of polyphenols [37], which explains a higher a* value compared to GC, but lower than GCGB and GCGO. However, from day 20 to the end of storage, GC exhibited the highest (*p* < 0.05) a* values among the treatments. At the same time, GCGB presented negative values, likely related to the oxidation/degradation of cachaça pigments or cofactors.

The parameter b* concerns the degree of blueness (from −100 to 0) and yellowness (from 0 to 70). All artisanal goat cheeses had a yellowish coloration, with no differences (*p* > 0.05) between samples at the beginning and end of storage (days 0 and 60). A similar pattern was observed throughout cold storage, with a slight (*p* < 0.05) increase (GCS) or stabilization (*p* > 0.05) (GC, GCGO, and GCGB) of b* values. This behavior suggests that cachaça immersion did not distinctly influence b* values throughout storage, which is a positive effect since the characteristic yellowish color of *coalho* cheese is highly valued by consumers from all parts of Brazil as an indicator of acceptability [32].

The parameter Chroma (C*) can be defined as the color intensity of the sample, ranging from dull (0) to vivid (60). No difference (*p* > 0.05) in C* values was observed among treatments for most of the storage. The exceptions were on day 10, where GC and GCGO presented lower (*p* < 0.05) intense color, and on day 50, where GC and GCS showed the highest (*p* < 0.05) color intensity among treatments. This result can be related to b* values (r = +0.999, *p* < 0.0001), where GC and GCGO revealed the lowest values on day 10, and the highest values were reported for GC and GCS on day 50. At the end of the storage period, C* values became similar (*p* > 0.05) for all treatments. Overall, all samples were considered to have low mean color intensity.

The hue angle (*h°*) corresponds to an angular position around a color coordinate chart describing the dimension of the color that is readily perceived by direct observation of the sample. On day 0 of storage, the cachaça-immersed treatments had lower (*p* < 0.05) *h°* values in the range of 85° to 86°, compared to GC (88°), indicating the predominance of yellow color in all treatments, which is expected for this product. During the middle storage period (from day 20 to 50), GC exhibited the lowest (*p* < 0.05) hue values among the treatments (84°~85°), indicating the tendency to reach a warmer yellow hue, corresponding to the higher (*p* < 0.05) a* values of GC observed from day 20 to 50. This pattern is corroborated by an inverse correlation between *h°* and a* (r = −0.979, *p* < 0.0001). In addition, the hue values of the artisanal cachaça-immersed cheeses tended to increase during storage towards a cool yellowish coloration, which is also consistent with the lower a* values reported for these treatments. Nevertheless, GCGB showed the highest (*p* < 0.05) hue value of 93° on the last day of storage, indicating an ivory-yellowish coloration of the samples [38]. Therefore, it is plausible to argue that a* mainly contributed to the variation in color characteristics of cachaça-immersed goat *coalho* cheese, which could lead to either acceptance or rejection of these products.

Finally, the total color difference (ΔE*) represents the variations in color between the freshly prepared treatments (day 0) and the subsequent samples, where ΔE* = 0 means that the color of the treatment is identical to the color of their freshly prepared counterpart. In addition, the ΔE* results can be classified as indistinct differences to the untrained human eye (Δ*E* < 3) and distinct differences to the human eye (Δ*E* > 3) [39]. No difference (*p* > 0.05) was observed among the treatments on days 10 and 60. However, GC presented the highest ΔE* values on days 20, 30, 40, and 50, indicating lower color stability of the control treatment. On the other hand, GCGO had the lowest ΔE* values on days 10, 20, 30, and 60, suggesting a more stable color of this cheese than the other immersed treatments.

### 3.4. Instrumental Texture

The texture parameter of artisanal goat cheeses results is shown in Table 3. On day 0 of storage, GC was the softest (*p* < 0.05) sample among all treatments, suggesting a hardening effect after cachaça-immersion. Thus, the lower hardness values of GC on day 0 can be attributed to a more significant weakening of the casein complex due to increased proteolysis during ripening, whether through the action of the residual rennet enzymes, the starter culture, or the milk background microbiota. The residual enzymes present in the coagulant added during cheese manufacturing are reported to have the primary effect on the initial proteolysis of artisanal goat *coalho* cheese. In contrast, the microbial proteolytic activity during ripening has a secondary effect [40].

Ethanol can directly affect rennet coagulation by promoting faster destabilization and aggregation of milk caseins [41]. However, casein was solubilized during rennet-coagulation, and cachaça-immersion occurred after coagulation was complete. Therefore, it is doubtful to affirm that ethanol contact with the cheese would promote main changes in the interaction between the casein matrix and the already aggregated micelles, leading to possible changes in the textural properties of pressed artisanal semi-hard cheeses. Therefore, we hypothesize that proteolysis in GCGO, GCGB, and GCS was weaker at the beginning of storage because ethanol slowed the residual coagulant enzyme activity during ripening, as ethanol is already known to considerably slow rennet enzyme proteolytic activity [42].

Moreover, the increased hardness values observed in GCGO and GCS at day 0 may be related to the lowest initial concentration of the *Lactococcus* spp. starter culture (Table 1), possibly injured during ripening, affecting its proteolytic activity or the pH reduction due to inefficient lactic acid production. In fact, pH is an important parameter for cheese hardness. Low pH values decrease the electrostatic repulsion between caseins, leading to increased casein-casein interactions and, consequently, higher hardness [43]. Comparable hardness results (around 600) were described for goat *coalho* cheese inoculated with the same starter culture used in this study, where the authors reported a non-significant influence of bacterial metabolism on cheese proteolysis due to the short ripening period applied (24 h) [12]. In contrast, the higher initial counts of *Lactococcus* spp. observed in GC and GCGB may have helped increase the cheese softening since the starter culture used in this study presents great proteolytic activity and lactic acid production [44].

No difference (*p* > 0.05) in hardness was observed in GCGO and GCS during storage. While GCGB and GC presented fluctuating values throughout the storage, with a tendency to increase. During most of the storage period, hardness values were similar in all treatments, but on day 60, the cachaça-immersed treatments had higher hardness values than GC. Increased hardness values in goat cheese can be related to increased syneresis and decreased moisture content caused by the natural shrinkage of the casein network during storage [45], which is more likely to happen in the presence of a dehydration agent [46] such as ethanol. On the other hand, a progressive increase in soluble calcium (from 75 to 168 mg/100 g) and a decrease in pH (from 6.81 to 5.23) was reported during 40 days of refrigerated storage of goat *coalho* cheeses, which was related to increased proteolysis [14]. Therefore, the fluctuating hardness values reported herein can be explained by the mutual occurrence of proteolysis and syneresis in artisanal goat *coalho* cheeses [47].

No difference (*p* > 0.05) was observed among cohesiveness results in artisanal goat *coalho* cheeses on day 0. During most of the storage, GC exhibited higher cohesiveness, with few variations. In fact, higher cohesiveness values are usually associated with higher hardness values caused by a pH decrease that leads to increased calcium solubilization and the straightening of the casein-casein complexes [34], which was not corroborated by our findings. At the end of storage, however, GCS and GCGB had the lowest (*p* < 0.05) cohesiveness values, which generally indicated an inverse relationship between cohesiveness and hardness, also seen in different storage moments. This fact may be related to the casein-complex structure composed of rigid but also very soft areas that are more accessible to hydrolysis by peptidases, which makes the cleavage sites’ accessibility uncertain [48] and leads to different cohesiveness results around cheese blocks.

Gumminess showed a similar trend to hardness, with GCGO and GCS having higher values at day 0 (*p* < 0.05), indicating a rubberier texture of these treatments compared to GC and GCGB. Gumminess was generally constant during storage, with a tendency to increase in the last days. In contrast, GCGB showed lower gumminess values in the first 50 days of storage compared to the other treatments, which may affect consumer acceptance since the rubbery texture is a desirable characteristic of *coalho* cheeses [32]. Subsequently, the gumminess values of GCGB increased on day 60 and showed the highest value (*p* < 0.05), which can also be related to the high hardness value of this sample on day 60.

The chewiness values presented no significant difference on day 0 (*p* > 0.05), regardless of cheese treatment, although GCS and GC had the highest values. Moreover, throughout the storage, the chewiness trend behavior was similar to that of hardness and gumminess, and, with few exceptions, GC had higher chewiness values among the treatments, followed by GCGO. On the last day of storage, GCGB exhibited the highest (*p* < 0.05) and GCS the lowest (*p* < 0.05) chewiness value, which was also observed for gumminess (r = +0.755, *p* < 0.0001). The same relationship between chewiness and gumminess and their association with hardness was described for goat *Minas frescal* cheese [28]. Finally, the texture values described herein are consistent with the results reported throughout the years for goat *coalho* cheeses, with high hardness, chewiness, and cohesiveness being appreciated by consumers [12,32,49,50].

### 3.5. Acceptance and Purchase Intention

The sensory scores of individual treatments for appearance, color, aroma, flavor, texture, and overall impression are presented in Table 4. The sensory evaluation happened between days 0 and 10 of storage. All treatments presented good sensory acceptance (scores ranging from 6.05 to 7.19—“like slightly” to “like moderately”), except for GCGB, which scored 5.99 (“neither like nor dislike”) in flavor attribute, differing (*p* < 0.05) from the other treatments. This treatment also presented lower (*p* < 0.05) scores for appearance and color compared to GC. This pattern is directly attributed to the gold cachaça stored in balsam casks, since we reported inverse correlations of color and a* (r = −0.961, *p* = 0.039), as well as appearance and LAB (r = -0.957, *p* = 0.043) and a* (r = −0.971, *p* = 0.029), suggesting that this cachaça variety increased the perception of yellow, and that consumers prefer cheeses with cool yellow tones to warmer tones, which represents a limiting factor for the use of this cachaça variety in artisanal cheese immersion (Table S2).

Finally, the aroma attribute was the only parameter not influenced by cachaça immersion (*p* > 0.05), which showed general acceptability. Regarding texture and overall impression, GC and GCGO presented the highest scores among the other treatments. Finally, concerning the purchase intention (Table 4), all treatments scored around 3 (“might or might not buy”). Overall, GC and GCGO presented the highest scores, indicating that consumers are more disposed to buy these two treatments over the others.

Although all cheeses presented good acceptance regardless of treatment, immersion can add value to the cheese, provide greater diversity in the market, and increase the acceptance of goat dairy products depending on the ingredient used. Although the cachaça-immersion did not lead to significant improvements in the sensory quality of the goat *coalho* cheese, it is worth noting that all treatments had similar acceptance and purchase intention among the panelists. This fact suggests that the artisanal cachaça-immersed goat cheese has the potential to be a viable alternative product for small-scale producers and family farming, as it can offer a unique and distinctive flavor that sets it apart from traditional goat *coalho* cheese. It is essential to consider that small-scale producers and family farming often struggle to compete in the market with larger, industrial producers, who can offer lower prices due to economies of scale [51]. Offering unique and differentiated products, such as cachaça-immersed goat *coalho* cheese, can provide these producers with a competitive advantage and help to diversify their product portfolio, ultimately contributing to the sustainability of their businesses.

In this context, when comparing the cachaça-immersed treatments, the aged cachaça granted more promising results than the unaged variations since GCGO obtained higher mean scores than GCGB and GCS regarding flavor, texture, overall impression, and purchase intention. Despite the scarcity of recent sensory studies, these results show a preference concerning the aged gold cachaça by consumers, which is consistent with reported studies [18,52].

## 4. Conclusions

The cachaça-immersion had no significant impact on the physicochemical properties of artisanal goat coalho cheese, which remained within the limits established by the legislation. Furthermore, starter culture viability was maintained, indicating that this method can be considered technologically feasible for small-scale cheesemakers. However, the color of the cheese was influenced by the type of cachaça, while the contact of ethanol with the cheese matrix led to a hardening effect that impacted consumer acceptance. Despite the lack of significant improvement in sensory attributes, our study suggests that the immersion of artisanal goat *coalho* cheese in gold cachaça aged in oak casks presents an opportunity for small-scale producers to introduce new products with unique sensory characteristics. However, further studies should investigate differences in immersion times and their effects concerning ethanol interaction with the casein-casein complex.

## Figures and Tables

**Table 1:** Estimated primary growth parameters (means ± standard deviation) of lactic acid bacteria (LAB) and *Lactococcus* spp. in artisanal goat *coalho* cheeses immersed in cachaça during 60 days of storage. Parameters were calculated using the DMFit tool from the ComBase software (browser.combase.cc/DMFit.aspx) based on the equations described by Baranyi and Roberts [24].

Bacterial Group	Treatments	*y*_0_ (log CFU/g)	*y_max_* (log CFU/g)	*µ_max_* (log CFU/g/h)	λ (h)	R^2^
LAB	GC	5.835 ± 0.46 ^A^	11.723 ± 0.16 ^A^	0.004 ± 0.00 ^A^	128.94 ± 1.71 ^B^	0.976
	GCCO	6.316 ± 0.33 ^A^	11.141 ± 0.72 ^A^	0.009 ± 0.01 ^A^	617.60 ± 70.25 ^A^	0.985
	GCGB	6.510 ± 0.42 ^A^	10.448 ± 0.56 ^A^	0.004 ± 0.00 ^A^	208.67 ± 1.16 ^B^	0.991
	GCS	6.519 ± 0.32 ^A^	11.766 ± 0.43 ^A^	0.006 ± 0.00 ^A^	637.68 ± 51.14 ^A^	0.972
*Lactococcus* spp.	GC	7.099 ± 0.08 ^A^	12.085 ± 0.96 ^A^	0.008 ± 0.00 ^A^	661.45 ± 11.73 ^A^	0.965
	GCCO	5.594 ± 0.37 ^C^	11.818 ± 0.11 ^A^	0.013 ± 0.00 ^A^	601.35 ± 65.32 ^A^	0.997
	GCGB	6.836 ± 0.05 ^A^	11.246 ± 0.46 ^A^	0.010 ± 0.00 ^A^	637.53 ± 23.43 ^A^	0.989
	GCS	6.161 ± 0.07 ^B^	12.102 ± 0.31 ^A^	0.012 ± 0.01 ^A^	658.74 ± 96.46 ^A^	0.989

^A–C^ Different uppercase superscripts in the same column indicate significant differences regarding treatments (*p* < 0.05); *y*_0_ = initial cell concentration (log CFU/g), *y_max_* = maximum cell concentration (log CFU/g), *µ_max_* = maximum growth rate (log CFU/g.h^−1^), λ = lag phase time (hour).

**Table 2:** Instrumental color parameters (means ± standard deviation) of artisanal goat *coalho* cheeses immersed in cachaça during 60 days of storage.

Parameter ^1^	Treatments	Storage Period (Days)						
		0	10	20	30	40	50	60
L*	GC	85.98 ± 1.10^aA^	86.24 ± 0.57 ^aB^	85.26 ± 1.74 ^abB^	82.91 ± 1.74 ^bcC^	80.70 ± 0.46 ^cB^	80.30 ± 0.92 ^cA^	80.18 ± 1.55 ^cA^
	GCCO	86.39 ± 0.76 ^aA^	86.48 ± 0.11 ^aB^	85.02 ± 0.81 ^abB^	85.72 ± 0.37 ^aAB^	84.44 ± 1.48 ^abA^	83.03 ± 2.37 ^abA^	81.25 ± 2.77 ^bA^
	GCGB	86.56 ± 0.79 ^bcdA^	88.78 ± 0.51 ^aA^	88.39 ± 0.36 ^abA^	87.12 ± 0.88 ^abcA^	86.18 ± 0.28 ^cdA^	84.63 ± 0.46 ^dA^	80.11 ± 1.16 ^eA^
	GCS	88.09 ± 1.22 ^aA^	86.53 ± 0.47 ^abB^	84.31 ± 1.31 ^abB^	85.17 ± 0.58 ^abB^	85.97 ± 0.84 ^abA^	82.87 ± 3.69 ^bA^	84.09 ± 0.87 ^abA^
a*	GC	0.30 ± 0.03 ^dC^	0.59 ± 0.06 ^cB^	1.17 ± 0.14 ^bA^	0.97 ± 0.08 ^bA^	1.13 ± 0.03 ^bA^	1.50 ± 0.20 ^aA^	0.37 ± 0.03 ^cdA^
	GCCO	0.65 ± 0.07 ^aB^	0.63 ± 0.09 ^abB^	0.35 ± 0.05 ^deC^	0.44 ± 0.04 ^cdC^	0.56 ± 0.01 ^abcB^	0.46 ± 0.12 ^bcdC^	0.21 ± 0.01 ^eB^
	GCGB	0.96 ± 0.02 ^aA^	0.83 ± 0.02 ^aA^	0.64 ± 0.09 ^bB^	0.61 ± 0.04 ^bB^	0.56 ± 0.07 ^bB^	0.32 ± 0.04 ^cC^	−0.60 ± 0.08 ^dC^
	GCS	0.63 ± 0.12 ^bB^	0.53 ± 0.04 ^bcB^	0.44 ± 0.04 ^bcBC^	0.41 ± 0.02 ^bcC^	0.37 ± 0.03 ^cC^	1.06 ± 0.15 ^aB^	0.37 ± 0.06 ^cA^
b*	GC	10.58 ± 0.47 ^bA^	10.32 ± 0.34 ^bB^	11.78 ± 0.71 ^bA^	12.80 ± 0.34 ^abA^	12.96 ± 1.85 ^abA^	15.47 ± 2.09 ^aA^	12.23 ± 1.67 ^abA^
	GCCO	11.84 ± 0.36 ^aA^	10.18 ± 0.35 ^aB^	11.76 ± 0.68 ^aA^	11.45 ± 0.86 ^aA^	10.68 ± 0.43 ^aA^	11.98 ± 1.02 ^aB^	11.43 ± 0.80 ^aA^
	GCGB	11.99 ± 1.36 ^aA^	11.47 ± 0.17 ^aA^	11.52 ± 0.25 ^aA^	11.68 ± 0.63 ^aA^	11.78 ± 0.07 ^aA^	12.45 ± 0.33 ^aAB^	12.54 ± 0.41 ^aA^
	GCS	10.24 ± 0.51 ^cA^	11.31 ± 0.12 ^bA^	11.56 ± 0.19 ^bA^	11.55 ± 0.23 ^bA^	11.54 ± 0.07 ^bA^	13.78 ± 0.28 ^aAB^	11.48 ± 0.36 ^bA^
C*	GC	10.58 ± 0.46 ^bA^	10.34 ± 0.34 ^bB^	11.84 ± 0.72 ^bA^	12.84 ± 0.33 ^abA^	13.01 ± 1.84 ^abA^	15.54 ± 2.10 ^aA^	12.23 ± 1.67 ^abA^
	GCCO	11.85 ± 0.36 ^aA^	10.20 ± 0.34 ^aB^	11.76 ± 0.68 ^aA^	11.46 ± 0.86 ^aA^	10.69 ± 0.43 ^aA^	11.99 ± 1.03 ^aB^	11.44 ± 0.80 ^aA^
	GCGB	12.03 ± 1.35 ^aA^	11.50 ± 0.17 ^aA^	11.53 ± 0.24 ^aA^	11.70 ± 0.62 ^aA^	11.80 ± 0.07 ^aA^	12.45 ± 0.33 ^aAB^	12.55 ± 0.40 ^aA^
	GCS	10.26 ± 0.52 ^cA^	11.33 ± 0.11 ^bA^	11.57 ± 0.19 ^bA^	11.56 ± 0.23 ^bA^	11.55 ± 0.07 ^bA^	13.82 ± 0.28 ^aA^	11.49 ± 0.36 ^bA^
*h°*	GC	88.37 ± 0.23 ^aA^	86.70 ± 0.39 ^bAB^	84.32 ± 0.50 ^dC^	85.67 ± 0.47 ^bcC^	84.96 ± 0.61 ^cdC^	84.46 ± 0.02 ^dC^	88.24 ± 0.15 ^aC^
	GCCO	86.87 ± 0.34 ^deB^	86.45 ± 0.59 ^eAB^	88.28 ± 0.33 ^abA^	87.78 ± 0.33 ^bcdAB^	87.00 ± 0.07 ^cdeB^	87.83 ± 0.39 ^bcA^	88.96 ± 0.08 ^aB^
	GCGB	85.36 ± 0.60 ^eC^	85.84 ± 0.14 ^deB^	86.81 ± 0.49 ^cdB^	87.02 ± 0.27 ^cB^	87.26 ± 0.31 ^cAB^	88.51 ± 0.21 ^bA^	92.76 ± 0.42 ^aA^
	GCS	86.47 ± 0.57 ^bcBC^	87.33 ± 0.23 ^abA^	87.82 ± 0.17 ^aAB^	87.95 ± 0.07 ^aA^	88.18 ± 0.17 ^aA^	85.59 ± 0.65 ^cB^	88.17 ± 0.24 ^aC^
ΔE*	GC	NA ^2^	1.25 ± 0.91^cA^	2.61 ± 1.05 ^bcAB^	3.87 ± 1.51 ^bcA^	5.97 ± 1.71 ^abA^	7.77 ± 1.69 ^aA^	6.17 ± 1.36 ^abA^
	GCCO	NA	1.80 ± 0.24 ^aA^	1.56 ± 0.91 ^aB^	1.09 ± 0.63 ^aB^	2.40 ± 1.82 ^aAB^	3.54 ± 2.25 ^aAB^	5.23 ± 2.26 ^aA^
	GCGB	NA	2.51 ± 0.72 ^bA^	2.28 ± 0.59 ^bAB^	1.40 ± 0.79 ^bAB^	1.48 ± 0.55 ^bB^	2.29 ± 1.16 ^bB^	6.72 ± 1.84 ^aA^
	GCS	NA	2.04 ± 0.62 ^cA^	4.02 ± 0.70 ^abA^	3.24 ± 0.72 ^abAB^	2.79 ± 1.19 ^bAB^	6.47 ± 2.57 ^aAB^	4.25 ± 0.57 ^abA^

^a–e^ Different lowercase superscripts in the same row indicate significant differences regarding storage times (*p* < 0.05); ^A–C^ Different uppercase superscripts in the same column indicate significant differences regarding treatments (*p* < 0.05); ^1^ Indicators of lightness (L*), redness (a*), yellowness (b*), Chroma (C*), hue angle (*h°*), and total color variation (ΔE*). ^2^ NA = not available. Values for these treatments were used to calculate ΔE* for the other treatments during storage.

**Table 3:** Instrumental texture parameters (means ± standard deviation) of artisanal goat *coalho* cheeses immersed in cachaça during 60 days of storage.

Parameter	Treatments	Storage Period (Days)						
		0	10	20	30	40	50	60
Hardness (g)	GC	326.80 ± 20.78 ^cC^	669.44 ± 46.82 ^aA^	610.15 ± 71.55 ^abAB^	670.94 ± 69.83 ^aA^	627.97 ± 28.17 ^abA^	527.54 ± 55.49 ^bA^	495.67 ± 11.14 ^bB^
	GCCO	600.06 ± 86.46 ^aAB^	574.52 ± 83.77 ^aAB^	726.35 ± 58.53 ^aA^	623.74 ± 75.87 ^aA^	633.52 ± 48.58 ^aA^	567.06 ± 19.20 ^aA^	613.37 ± 80.05 ^aAB^
	GCGB	486.73 ± 59.75 ^bcB^	418.35 ± 39.61 ^cB^	471.62 ± 65.79 ^bcB^	556.96 ± 82.74 ^abcA^	600.78 ± 70.27 ^abA^	519.47 ± 54.09 ^bcA^	719.56 ± 45.51 ^aA^
	GCS	672.47 ± 25.05 ^aA^	628.64 ± 94.48 ^aA^	645.19 ± 99.71 ^aAB^	750.48 ± 74.24^aA^	771.76 ± 103.08 ^aA^	670.82 ± 92.40 ^aA^	672.95 ± 69.24 ^aA^
Cohesiveness	GC	0.94 ± 0.01 ^bcA^	0.98 ± 0.05 ^bcA^	0.92 ± 0.06 ^bcA^	0.83 ± 0.02 ^cB^	1.07 ± 0.11 ^abA^	0.94 ± 0.07 ^bcA^	1.20 ± 0.03 ^aA^
	GCCO	0.86 ± 0.06 ^abA^	0.87 ± 0.06 ^abAB^	0.72 ± 0.08 ^bB^	0.96 ± 0.04 ^aA^	0.80 ± 0.06 ^bB^	0.81 ± 0.05 ^abAB^	0.80 ± 0.04 ^bB^
	GCGB	0.89 ± 0.02 ^abA^	0.97 ± 0.06 ^aA^	0.83 ± 0.03 ^bcAB^	0.75 ± 0.02 ^cdB^	0.73 ± 0.02 ^dB^	0.75 ± 0.03 ^cdB^	0.78 ± 0.03 ^cdBC^
	GCS	0.83 ± 0.09 ^aA^	0.75 ± 0.04 ^aB^	0.73 ± 0.03 ^aB^	0.79 ± 0.07 ^aB^	0.73 ± 0.09 ^aB^	0.69 ± 0.04 ^aB^	0.68 ± 0.06 ^aC^
Gumminess (g)	GC	428.88 ± 4.52 ^dB^	672.33 ± 29.77 ^abA^	536.19 ± 35.26 ^bcdA^	546.88 ± 5.09 ^bcdA^	762.45 ± 116.08 ^aA^	502.04 ± 30.67 ^cdA^	571.89 ± 31.52 ^bcB^
	GCCO	513.48 ± 41.72 ^bA^	451.24 ± 39.98 ^bB^	508.59 ± 21.96 ^bA^	517.09 ± 28.29 ^bA^	497.55 ± 20.28 ^bBC^	468.39 ± 10.07 ^bA^	621.47 ± 11.80 ^aB^
	GCGB	398.76 ± 26.12 ^bB^	346.72 ± 41.36 ^bC^	336.47 ± 9.87 ^bB^	335.10 ± 8.81 ^bB^	352.02 ± 27.74 ^bC^	378.51 ± 40.11 ^bB^	817.36 ± 31.46 ^aA^
	GCS	437.97 ± 43.32 ^bcAB^	458.43 ± 36.29 ^abcB^	372.83 ± 21.74 ^cB^	521.33 ± 16.05 ^abA^	543.57 ± 45.15 ^aB^	434.46 ± 13.40 ^bcAB^	403.35 ± 51.38 ^cC^
Chewiness (g)	GC	4149.62 ± 464.57 ^dA^	7134.70 ± 1053.94 ^abA^	6398.09 ± 650.39 ^bcA^	5361.97 ± 756.73 ^bcdA^	8988.96 ± 563.27 ^aA^	4233.71 ± 373.64 ^dA^	4668.38 ± 669.98 ^cdAB^
	GCCO	3972.43 ± 570.73 ^bcA^	4941.72 ± 521.11 ^abB^	4791.45 ± 722.57 ^abB^	5545.21 ± 526.37 ^aA^	3873.27 ± 496.08 ^bcB^	3098.07 ± 415.51 ^cB^	3480.27 ± 354.39 ^bcBC^
	GCGB	3361.18 ± 351.34 ^bA^	3734.48 ± 539.67 ^bBC^	3046.91 ± 301.08 ^bcC^	3190.76 ± 400.28 ^bcB^	3179.45 ± 165.02 ^bcB^	2007.65 ± 4.65 ^cC^	5822.61 ± 857.47 ^aA^
	GCS	4705.68 ± 728.45 ^aA^	3179.86 ± 267.26 ^bcC^	2391.29 ± 190.73 ^cC^	4466.41 ± 677.78 ^abAB^	3428.05 ± 499.87 ^abcB^	2718.62 ± 349.33 ^cBC^	2678.35 ± 362.12 ^cC^

^a–d^ Different lowercase superscripts in the same row indicate significant differences regarding storage times (*p* < 0.05); ^A–C^ Different uppercase superscripts in the same column indicate significant differences regarding treatments (*p* < 0.05).

**Table 4:** Sensory acceptance and purchase intention of artisanal goat *coalho* cheeses immersed in cachaça.

Parameters ^1^		Treatments			
		GC	GCGO	GCGB	GCS
Sensory acceptance	Appearance	7.19 ± 1.57 ^A^	6.81 ± 1.75 ^AB^	6.39 ± 1.80 ^B^	6.96 ± 1.65 ^AB^
	Color	7.12 ± 1.50 ^A^	6.77 ± 1.71 ^AB^	6.41 ± 1.81 ^B^	6.94 ± 1.62 ^AB^
	Aroma	7.04 ± 1.55 ^A^	7.07 ± 1.48 ^A^	6.66 ± 1.46 ^A^	7.03 ± 1.30 ^A^
	Flavor	6.81 ± 1.81 ^A^	6.69 ± 1.90 ^A^	5.99 ± 1.94 ^B^	6.63 ± 1.68 ^A^
	Texture	7.13 ± 1.82 ^A^	6.86 ± 1.86 ^AB^	6.05 ± 2.11 ^C^	6.20 ± 2.05 ^BC^
	Overall impression	6.90 ± 1.73 ^A^	6.71 ± 1.76 ^A^	6.06 ± 1.78 ^B^	6.57 ± 1.55 ^AB^
Purchase intention		3.63 ± 1.06 ^A^	3.59 ± 1.20 ^A^	3.13 ± 1.11 ^B^	3.26 ± 1.02 ^AB^

^A–C^ Different uppercase superscripts in the same row indicate significant differences regarding treatments (*p* < 0.05); ^1^ Sensory acceptance was evaluated in a 9-point hedonic scale and purchase intention was evaluated in a 5-point hedonic scale.

## Data Availability

No new data were created or analyzed in this study. Data sharing is not applicable to this article.

## Supplementary Materials

The following supporting information can be downloaded at: https://www.mdpi.com/article/10.3390/foods12101945/s1, Table S1: Pearson’s correlation test between microbial count and physicochemical parameters in artisanal goat *coalho* cheeses immersed in cachaça and stored at 4 °C for 60 days; Table S2: Pearson’s correlation test between microbial and physicochemical variables with sensory quality scores in artisanal goat *coalho* cheeses immersed in cachaça and stored at 4 °C.
