# Supplementary material for: Artisanal Goat Coalho Cheese with Cachaça as Flavoring Agent
Source: Foods. 2023 May 10;12(10):1945. doi: 10.3390/foods12101945 (PMC10217206; doi:10.3390/foods12101945)
Supplement: Supplementary file 1 [file foods-12-01945-s001.zip › foods-2372400-supplementary.pdf]

Artisanal goat *coalho* cheese with cachaça as flavoring agent

Iuri Rosario, Carla Vieira, Luana Barreto, Nathália Monteiro, Rodrigo Moreira, Ana Paula Salim, Carini Lelis, Manuela Solca, Sergio Mano, Carlos Conte-Junior and Marion Costa

Table S1. Pearson's correlation test between microbial count and physicochemical parameters in artisanal goat *coalho* cheeses immersed in cachaça and stored at 4 °C during 60 days.

Parameter	Variable	r	*p*
Storage period	LAB	**0.958**	<0.0001
	Lactococcus spp.	**0.932**	<0.0001
	pH	-0.310	0.108
	*L**	**-0.747**	<0.0001
	*a**	-0.192	0.328
	*b**	0.506	0.006
	*C**	0.501	0.007
	h°	0.314	0.104
	Hardness	0.265	0.173
	Cohesiveness	-0.121	0.541
	Gumminess	0.297	0.125
	Chewiness	-0.113	0.567
LAB	Storage period	**0.958**	<0.0001
	Lactococcus spp.	**0.937**	<0.0001
	pH	0.357	0.062
	*L**	**-0.733**	<0.0001
	*a**	-0.050	0.800
	*b**	0.522	0.004
	*C**	0.519	0.005
	h°	0.166	0.400
	Hardness	0.101	0.608
	Cohesiveness	-0.031	0.875
	Gumminess	0.229	0.241
	Chewiness	-0.116	0.557
Lactococcus	Storage period	**0.932**	<0.0001
	LAB	**0.937**	<0.0001
	pH	0.356	0.063
	*L**	**-0.723**	<0.0001
	*a**	-0.130	0.510
	*b**	0.470	0.012
	*C**	0.466	0.012
	h°	0.240	0.219

	Hardness	0.062	0.755
	Cohesiveness	-0.021	0.916
	Gumminess	0.268	0.168
	Chewiness	-0.146	0.459
pH	Storage period	-0.310	0.108
	LAB	0.357	0.062
	Lactococcus spp.	0.356	0.063
	*L**	-0.553	0.002
	*a**	-0.140	0.477
	*b**	0.170	0.387
	*C**	0.166	0.399
	h°	0.180	0.360
	Hardness	-0.148	0.452
	Cohesiveness	0.646	0.0002
	Gumminess	0.367	0.055
	Chewiness	0.229	0.241
*L**	Storage period	**-0.747**	<0.0001
	LAB	**-0.733**	<0.0001
	Lactococcus spp.	**-0.723**	<0.0001
	pH	-0.553	0.002
	*a**	0.053	0.787
	*b**	-0.627	0.001
	*C**	-0.625	0.001
	h°	-0.196	0.319
	Hardness	-0.197	0.315
	Cohesiveness	-0.205	0.296
	Gumminess	-0.634	0.001
	Chewiness	-0.256	0.189
*a**	Storage period	-0.192	0.328
	LAB	-0.050	0.780
	Lactococcus spp.	-0.130	0.510
	pH	-0.192	0.328
	*L**	0.053	0.787
	*b**	0.416	0.028
	*C**	0.426	0.024
	h°	**-0.979**	<0.0001
	Hardness	-0.205	0.295
	Cohesiveness	0.270	0.166
	Gumminess	-0.235	0.229
	Chewiness	0.169	0.391
*b**	Storage period	0.506	0.006
	LAB	0.522	0.004
	Lactococcus spp.	0.470	0.012
	pH	0.170	0.387
	*L**	-0.627	0.001
	*a**	0.417	0.023
	*C**	**0.999**	<0.0001
	h°	-0.232	0.235

	Hardness	0.005	0.980
	Cohesiveness	0.050	0.801
	Gumminess	0.152	0.442
	Chewiness	-0.011	0.957
*C**	Storage period	0.501	0.007
	LAB	0.519	0.005
	Lactococcus spp.	0.466	0.012
	pH	0.166	0.399
	*L**	-0.625	0.001
	*a**	0.426	0.024
	*b**	**0.999**	<0.0001
	h°	-0.243	0.213
	Hardness	0.004	0.982
	Cohesiveness	0.053	0.788
	Gumminess	0.152	0.440
	Chewiness	-0.005	0.982
h°	Storage period	0.314	0.104
	LAB	0.166	0.400
	Lactococcus spp.	0.240	0.219
	pH	0.180	0.360
	*L**	-0.195	0.319
	*a**	-0.979	<0.0001
	*b**	-0.232	0.235
	*C**	-0.243	0.213
	Hardness	0.214	0.273
	Cohesiveness	-0.283	0.144
	Gumminess	0.278	0.152
	Chewiness	-0.195	0.320
Hardness (g)	Storage period	0.265	0.173
	LAB	0.101	0.608
	Lactococcus spp.	0.062	0.755
	pH	-0.148	0.452
	*L**	-0.197	0.315
	*a**	-0.205	0.295
	*b**	0.005	0.980
	*C**	0.004	0.982
	h°	0.214	0.273
	Cohesiveness	-0.456	0.015
	Gumminess	0.437	0.020
	Chewiness	0.206	0.294
Cohesiveness	Storage period	-0.121	0.541
	LAB	-0.031	0.875
	Lactococcus spp.	-0.021	0.916
	pH	0.646	0.0002
	*L**	-0.205	0.296
	*a**	0.270	0.166
	*b**	0.050	0.801
	*C**	0.053	0.788

	h°	-0.283	0.144
	Hardness	-0.456	0.015
	Gumminess	0.355	0.064
	Chewiness	0.607	0.001
Gumminess (g)	Storage period	0.297	0.125
	LAB	0.229	0.241
	Lactococcus spp.	0.268	0.168
	pH	0.367	0.055
	*L**	-0.634	0.001
	*a**	-0.235	0.229
	*b**	0.152	0.442
	*C**	0.152	0.440
	h°	0.278	0.152
	Hardness	0.437	0.020
	Cohesiveness	0.355	0.064
	Chewiness	**0.755**	<0.0001
Chewiness (g)	Storage period	-0.113	0.567
	LAB	-0.116	0.557
	Lactococcus spp.	-0.146	0.459
	pH	0.229	0.241
	*L**	-0.256	0.189
	*a**	0.169	0.391
	*b**	-0.011	0.957
	*C**	-0.005	0.982
	h°	-0.195	0.320
	Hardness	0.206	0.294
	Cohesiveness	0.607	0.001
	Gumminess	**0.755**	<0.0001

LAB: lactic acid bacteria; *L**: lightness; *a**: redness; *b**: yellowness; *C**: chroma; *h*°: hue angle; r: Pearson´s correlation coefficient; *p*: probability value. Values in bold indicate strong (≥ 0.7) correlation between the variables.

Table S2. Pearson's correlation test between microbial and physicochemical variables with sensory quality scores in artisanal goat *coalho* cheeses immersed in cachaça and stored at 4 °C.

Sensory parameter	Variable	R	*p*
Appearance	LAB	**-0.957**	0.043
	Lactococcus	-0.024	0.976
	*L**	-0.013	0.987
	*b**	-0.794	0.206
	*a**	**-0.971**	0.029
	*C**	-0.804	0.196
	h°	0.919	0.081
	Hardness	-0.241	0.759
	Cohesiveness	0.234	0.766
	Gumminess	0.249	0.751
	Chewiness	0.761	0.239
Color	LAB	**-0.954**	0.047
	Lactococcus	-0.018	0.982
	*L**	0.031	0.969
	*b**	-0.823	0.177
	*a**	**-0.961**	0.039
	*C**	-0.832	0.168
	h°	0.900	0.100
	Hardness	-0.219	0.781
	Cohesiveness	0.209	0.791
	Gumminess	0.222	0.778
	Chewiness	0.785	0.215
Aroma	LAB	-0.687	0.313
	Lactococcus	-0.529	0.471
	*L**	0.093	0.907
	*b**	-0.555	0.445
	*a**	-0.784	0.217
	*C**	-0.567	0.433
	h°	0.746	0.254
	Hardness	0.160	0.840
	Cohesiveness	-0.143	0.857
	Gumminess	0.691	0.309
	Chewiness	0.781	0.219
Flavor	LAB	-0.822	0.178
	Lactococcus	-0.338	0.662
	*L**	-0.036	0.964
	*b**	-0.610	0.390
	*a**	-0.899	0.101
	*C**	-0.623	0.377
	h°	0.871	0.129
	Hardness	-0.049	0.951
	Cohesiveness	0.062	0.938
	Gumminess	0.571	0.429
	Chewiness	0.742	0.259

Texture	LAB	-0.743	0.257
	Lactococcus	-0.034	0.966
	*L**	-0.650	0.350
	*b**	-0.160	0.840
	*a**	-0.837	0.164
	*C**	-0.176	0.824
	h°	0.915	0.085
	Hardness	-0.530	0.470
	Cohesiveness	0.564	0.436
	Gumminess	0.495	0.506
	Chewiness	0.173	0.827
Overall impression	LAB	-0.867	0.133
	Lactococcus	-0.220	0.780
	*L**	-0.208	0.792
	*b**	-0.546	0.454
	*a**	-0.942	0.058
	*C**	-0.560	0.440
	h°	0.939	0.061
	Hardness	-0.219	0.782
	Cohesiveness	0.235	0.765
	Gumminess	0.529	0.471
	Chewiness	0.624	0.376
Purchase intention	LAB	-0.695	0.305
	Lactococcus	-0.207	0.793
	*L**	-0.561	0.439
	*b**	-0.153	0.847
	*a**	-0.811	0.190
	*C**	-0.170	0.830
	h°	0.882	0.118
	Hardness	-0.374	0.626
	Cohesiveness	0.413	0.587
	Gumminess	0.633	0.367
	Chewiness	0.251	0.750

LAB: lactic acid bacteria; *L*:* lightness; *a*:* redness; *b*:* yellowness; *C**: chroma; *h*°: hue angle; r: Pearson´s correlation coefficient; *p*: probability value. Values in bold indicate strong (≥ 0.7) correlation between the variables.
